# Examining the Effectiveness of Ayres Sensory Integration® Intervention for Children With Developmental Coordination Disorder in Improving Motor Coordination and Daily Activity Function: A Randomized Controlled Trial

**DOI:** 10.7759/cureus.76971

**Published:** 2025-01-05

**Authors:** Yoko Yamanishi, Yasushi Orita, Mika Nagayoshi, Rie Nishimura, Tamae Shinjyo, Kumiko Masuda, Yuko Hayashi, Akio Nakai, Akira Imamura, L. Diane Parham, Ryoichiro Iwanaga

**Affiliations:** 1 Department of Occupational Therapy Science, Graduate School of Biomedical Sciences, Nagasaki University, Nagasaki, JPN; 2 Department of Occupational Therapy, Faculty of Health and Welfare, Prefectural University of Hiroshima, Mihara, JPN; 3 Department of Occupational Therapy, Graduate School of Human Health Sciences, Tokyo Metropolitan University, Arakawa, JPN; 4 Institute for Child Development, General Incorporated Association of AQUA, Ginoza, JPN; 5 Department of Occupational Therapy, Fukuyama Support Center of Development and Care for Children, Fukuyama, JPN; 6 Department of Occupational Therapy, Kurashige Pediatric Clinic, Kitakyushu, JPN; 7 Faculty of Health and Welfare, Medical Center, Prefectural University of Hiroshima, Mihara, JPN; 8 Research Institute for Education and Graduate School of Clinical Education, Mukogawa Women's University, Nishinomiya, JPN; 9 Department of Pediatrics, Occupational Therapy Graduate Program, University of New Mexico, Albuquerque, USA

**Keywords:** ayres sensory integration, children, daily life, developmental coordination disorder, goal attainment, intervention, rct, sensory integration

## Abstract

Background

Ayres Sensory Integration^®^ (ASI) intervention focuses on developing sensory processing abilities to improve motor coordination, executive functions, participation, and satisfaction in everyday activities. No well-designed research studies have addressed clearly the effectiveness of ASI intervention for children with developmental coordination disorder (DCD) even though ASI intervention was effective for children with autism spectrum disorders.

Methods and procedures

Seventeen children with DCD (aged 4-8.5 years old) were randomly assigned to either an intervention or a control group. ASI intervention was provided to the intervention group twice a week for 10 weeks. In addition to participants’ goals in daily activities, sensory integration abilities and motor coordination were assessed before and after the intervention.

Outcomes and results

The split-plot factorial design demonstrated significant time × group interaction in the total score (F (1, 15) = 7.651, p = 0.014, partial η^2^= 0.338) and balance score (F (1, 15) = 11.163, p = 0.004, partial η^2^ = 0.427) of the Movement Assessment Battery for Children-Second Edition (MABC-2), with significant differences in simple main effects before and after intervention for the intervention group. The post-intervention Goal Attainment Scale (GAS) score showed a significant difference in the time × group interaction (F (1, 15) = 15.662, p = 0.001, partial η^2^ = 0.511) and a simple main effect in the intervention group.

Conclusions

A short-term, intensive ASI intervention improves motor performance, coordination, and daily activities function in children with DCD.

## Introduction

Developmental coordination disorder (DCD) is a neurodevelopmental disorder that affects 5-8% of school-aged children [1]. DCD is a condition in which a child's motor coordination performs below the level of children of the same age and that affects the activities of daily living. For instance, activities of daily living (such as dressing and eating), school subjects (such as writing and physical education), music, and playing games (such as ball throwing and football) affect the child in various situations, with difficulties leading to a decrease in self-esteem [2,3]. In addition, 87% of children with autism spectrum disorder (ASD) [4] and 73% of children with attention-deficit-hyperactivity disorder (ADHD) [5] have coexisting motor difficulties. Those coordination problems may be not recognized enough as one of the DCD symptoms in neurodevelopmental disorders. Therefore, motor coordination problems could be often overlooked at home or school, particularly when no significant behavioral or emotional challenges exist. Children with DCD are more likely to be maladjusted at school and have low life satisfaction, and this is related to the quality of life [6,7]. Hence, motor coordination difficulties should not be overlooked when families and teachers seek support for their children whose movement skills are immature or ineffective.

One of the neurological mechanisms of DCD is internal model deficit. In the motor learning process, the results of the actual outcome are feedbacked, and the motor planning is modified based on the error signals between the predicted and actual results. A model of movement stored in the brain is called the internal model. Children with DCD have a tendency to lose this internal model and have difficulty reflecting in action immediately [3,8]. Even though body schema and body image are important as precursors to the planning and execution of movement, children with DCD are in a deficit of the plasticity of the body schema [9], in addition to body schema deficit leading to inefficient body image with DCD children [10]. The mirror neuron system (MNS) is a frontoparietal neural network that activates when a person is observing, imitating actions, imaging, or executing. However, children with DCD are impaired with this MNS function [11], which states that underlying difficulties with sensory and motor information processing may account for DCD [12]. Furthermore, the execution of movement requires the combined information of both reflections to the environment stimuli and integration of various senses, particularly vestibular, proprioception (muscle stretch and joint reception), and vision. Although previous studies reported that children with DCD have sensory processing problems as compared with typically developing children, the relationship between sensory processing and motor abilities remains an issue to be solved [13-15].

As an intervention approach, the activity-oriented approach, as represented by the Cognitive Orientation to Daily Occupational Performance (CO-OP) approach [16], which focuses on the use of cognitive strategies to guide goal-directed actions, was developed specifically for children with DCD. It is recommended in international guidelines as an effective intervention for children with DCD, as well as children with other conditions that interfere with the intentional planning of movements [3]. On the other hand, as an approach for children with motor coordination difficulties, the process-oriented approach focuses on improving the neural substrates of skilled movement and includes Ayres Sensory Integration® (ASI) intervention, which has been conventionally held. ASI intervention focuses on the child’s involvement in independent sensory-motor play, with therapist guidance to encourage the child’s ideas while ensuring safety [17]. This intervention is implemented in a play environment, usually within an indoor therapy gym or playroom, that provides opportunities for a variety of sensory-motor activities and challenges while the therapist ensures safety. ASI intervention aims to improve and enhance the neural basis of the child's sensory processing to improve functional abilities such as postural responses, planning of actions, ability to focus attention, and ability to sustain concentration. The therapy is implemented through therapist interactions with the child and is based on play so that the child can actively participate in activities that they select and enjoy. Gradually increasing the activity challenges is crucial so that the play activities in therapy become the “just right challenge” for each child, i.e., not so difficult that the child cannot be successful, but not so easy that it requires little or no effort from the child [18]. Furthermore, the therapy process aims to support the child’s development of self-confidence, motivation and willingness, achievement, and competence in everyday life activities, including self-care skills and chores at home, as well as academic performance and peer relationships at school.

Several recent high-quality randomized controlled trials (RCTs), as well as a systematic review, have yielded positive outcomes of ASI intervention for children with ASD [19,20]. Generally, these studies have indicated that ASI intervention improves sensory processing and functional movement abilities [21]. Additionally, ASI intervention outcomes have included improvements in the daily living skills of children with sensory integration challenges. The best practice in goal setting involves collaboration among the therapist, the child, and the child’s family to decide on short-term as well as long-term goals that are likely to lead to desirable outcomes that are meaningful and achievable.

Although this growing body of studies indicates that ASI intervention is effective for children with ASD, it has not been defined how the movement challenges of children with DCD may affect their participation in solitary play, social play, schoolwork, household chores, and other activities. On the other hand, motor difficulties are co-occurring with ASD [3,4], and improvement of motor difficulties through previous intervention studies could be related to ASD conditions. The effectiveness of sensory processing intervention in children with DCD or children with sensory-based motor difficulties is expected [22,23], while reports of interventions and evidence-based best practices are limited or unavailable, and the effectiveness of ASI is uncertain for DCD children with and without ASD, and other developmental conditions. This research was conducted to examine the effectiveness of ASI intervention for children with DCD. The specific purposes of the study were to determine the effectiveness (if any) of ASI intervention in improving motor coordination and to capture changes in the children's participation in daily life activities. Ultimately, our goal is to contribute knowledge of evidence-based practices that will support the quality of life for children with DCD.

## Materials and methods

Study design

This research involves an intervention study utilizing a randomized controlled trial (RCT) with intervention and control groups (1:1). The study was conducted in accordance with the Declaration of Helsinki, and the protocol was approved by the Ethics Committee of the Prefectural University of Hiroshima (project identification code: 18MH016-01; dated: 28th June 2019) and the Ethics Committee of the Nagasaki University Graduate School of Biomedical Sciences (project identification code: 1909120; dated: 1st November 2019). The study was registered in the University Medical Information Network (UMIN) Clinical Trials Registry (UMIN: 000037263).

Participants' characteristic

Participant Criteria

Prior to participation in this study, participants were required to meet the following eligibility criteria: (i) difficulties with coordinating movements in daily life; (ii) being diagnosed with DCD by a pediatrician using the diagnostic criteria of the Diagnostic and Statistical Manual of Mental Disorders, Fifth Edition (DSM-5) [24]; (iii) aged between four years and eight years and six months; (iv) at least one of the four primary scores of the Movement Assessment Battery for Children-Second Edition (MABC-2) [25] falling below the 16th percentile (manual dexterity, aiming and catching, balance, and total score); and (v) three or more items that fall at or below -0.7 standard deviation (SD) ranges, from the 16 items in the somatosensory perception, motor performance, visual perception, and others scales of the Southern California Sensory Integration Tests (SCSIT) [26]. Age criteria were decided according to previous studies showing that symptoms of DCD become apparent between the ages of four and five [3] and ASI intervention criteria [19,27,28]. Children were excluded from the study if they met one or more of the following exclusion criteria: (i) confirmed diagnosis of intellectual disability, with a full-scale intelligence quotient (IQ) score of <70 on the Wechsler Intelligence Scale for Children-Fourth Edition (WISC-IV) [29], or developmental quotients (DQ) <70 on the Kyoto Scale of Psychological Development (KSPD) [30]; (ii) confirmed diagnosis of cerebral palsy or other neuromuscular condition; (iii) history of previously receiving ASI intervention; (iv) unable to consistently attend therapy twice a week, resulting in less than 960 minutes (80% of total sessions) of intervention attended. We had estimated total therapy time based on previous evidenced studies [19,31,32] that a minimum of 840 minutes total therapy time was sufficient to get effective results.

Participant Recruitment

Posters were distributed to child development support centers and daycare centers to inform parents about the study. Child development support centers serve preschool children, and daycare is one of the welfare services for school-aged children in Japan. These are places where children can receive individual or group services to help them develop physical and mental functions such as life skills, social skills, and literacy skills as they progress through life stages. Parents who wished to participate were given an in-person and written explanation about the study and were invited and asked to participate. We verbally explained research contents to children, using simple written and illustrated ascent documents. As an ethical consideration, the control group was ensured to have an opportunity of the same structure intervention (e.g., time and frequency) as the intervention group after the first intervention period ended. Both groups continued to receive social services and attended their child care center and school. All participants used the same day-service organization, but they did not receive individualized support to improve their physical and psychological functioning.

A priori power analysis was conducted using G*Power 3.1 (Heinrich Heine University Düsseldorf, Düsseldorf, Germany). Considering a large effect size of partial η^2^ = 0.14 (Cohen’s f = 0.4), a significance level of = 0.05, and a power of 80% on an analysis of variance for split-plot factorial design, the required sample size was calculated to be 16 participants. Assuming a 20% dropout rate, we decided to finalize the total sample size of 20. At the time of consent, each participant was assigned a subject identification number. Randomization was performed by the co-investigators using a computer program that assigned each subject to either the intervention group or the control group. The evaluators and interventionists who participated in the assessments and/or the randomization procedures were independent and blinded to the group to which each subject had been assigned. All pre- and post-intervention assessments of participants, including children and their parents, were conducted by the third author and the sixth author other than the interventionists.

Basic information about the characteristics of the participants

Kyoto Scale of Psychological Development (KSPD)

The KSPD [30] is widely used in Japan for developmental testing in infancy. The assessment is divided into three domains: cognitive-adaptive (C-A), language-social (L-S), and postural-motor (P-M). Scores are calculated for each domain, which contributes to the overall developmental quotients (DQ). The split-half reliabilities of each of the three scales are as follows: for the P-M, 0.980 for ages one to three; for the C-A, 0.931 for ages three to six; for the L-S, 0.906 for ages three to five [30]. A previous study reported that the DQ could estimate cognitive ability in children with ASD [33]. Therefore, in the present study, we used the DQ of the KSPD score to determine the developmental levels of preschool-age participants and school-age participants aged six years old.

Wechsler Intelligence Scale for Children-Fourth Edition (WISC-IV)

The WISC-IV [29] is one of the most widely used assessments of the neuropsychological development of children aged five years to 16 years and 11 months. This scale is standardized for Japanese children. The internal consistency was 0.86 to 0.95. [29]. The WISC-IV was used to assess the development of school-age participants and over. The WISC-IV Full-Scale Intelligence Quotient (FSIQ) scores were used in this study.

*Short* *Sensory Profile (SSP)*

The Japanese version of the SSP [34] is a caregiver or parent-reported questionnaire to assess children's sensory processing issues in daily life. There are a total of 38 items, divided into seven sections: tactile sensitivity, taste and smell sensitivity, motor sensitivity, hyporesponsiveness and sensation seeking, auditory filtering, hypoactivity and weakness, and visual and auditory sensitivity. The Japanese version of the SSP has been demonstrated to have comparable utility [35]. Parents scored the Japanese version of the SSP before and after the intervention.

Japanese Version of the ADHD-Rating Scale-IV (ADHD-RS) and Social Responsiveness Scale, Second Edition (SRS-2)

To clarify the extent of ADHD and ASD symptoms within our study participants, we used the Japanese version of the ADHD-RS [36] and the Japanese version of the SRS-2 [37]. Both assessments are questionnaires with parent rating scales. The Japanese version of the ADHD-RS consists of 18 items. The Cronbach’s alpha is for the two subscales of ADHD-RS and was found sufficient: 0.88 for inattentive and 0.85 for hyperactive-impulsive. The Japanese version of the SRS-2 consists of 65 items. The Cronbach’s alpha is over 0.918 [37]. These two scales have sufficient reliability. In this study, we used the total score of ADHD-RS and the T-score of SRS-2.

Primary outcome measure

Movement Assessment Battery for Children-Second Edition (MABC-2)

MABC-2 [25] was used to measure participants’ coordination of motor skills. MABC-2 is a gold standard assessment of coordinated movement and is divided into three age bands: three to six years old, seven to 10 years old, and 11 to 16 years old. Each age band consists of three domains, namely, manual dexterity (three tests), aiming and catching (two tests), and balance (three tests), with a total score calculated. MABC-2 was not standardized in Japan. However, internal consistency for Japanese children showed acceptable values (age bands: three to six years was 0.624 and seven to ten years was 0.602) [38,39]. In factorial validity, the theoretical three-component model was not fitted to Japanese children with an age band of three to six years. A total test score should be assigned to children aged three to six. Concurrent validity has also been demonstrated [25]. The test results are converted to standard scores with 10 being the average standard score and higher scores indicating better motor coordination skills. The MABC-2 was administered to all participants who were recruited in the study to determine the degree of severity of the motor difficulties. Subjects after the exclusion procedure were reassessed post intervention.

Goal Attainment Scaling (GAS)

Goal Attainment Scaling (GAS) [40] is an evaluation method in which initial goals are set in collaboration with the subject or with the primary caregiver, if the subject is a child, and individual subjects' goals are measured before and after the intervention (Appendix). GAS has been successfully used in ASI effectiveness studies [19,31,32]. Each goal was scored on a five-point scale, and achievement was measured after the intervention was completed. GAS is scored on a five-point scale ranging from -2 to +2, with -1 representing current ability and 0 representing goals expected to be achieved during the intervention period. The GAS score is calculated for each subject child by fitting a T-score to a formula [40]. A GAS-modified score of 50 indicates the predicted outcome. A score higher than 50 represents a better-than-predicted outcome, and a score lower than 50 indicates a less-than-predicted outcome. In this study, GAS goals are identified and set up as their child’s specific goals in collaboration with parents and an independent evaluator (occupational therapist), and the scores for each child as well as the average T-score for each group were calculated. As the participants in this study had motor coordination difficulties as their main symptom, the goals were based on “things related to movement such as cutting a paper or going up the stairs” and “things that reflect the child's intention and motivation” to confirm their achievements. We did not home-program for parents and children in relation to the goals. Children and parents live the same life as before participating in this study.

Secondary outcome measure

Southern California Sensory Integration Tests (SCSIT)

The SCSIT [26] was used to measure sensory integration function in participants who were identified as having motor coordination issues based on the results of the MABC-2 assessment. The SCSIT was established as a measure of sensory integration function and was later modified and published as the Sensory Integration and Praxis Tests (SIPT) [41]. In Japan, the SCSIT has been used for many years in sensory integration therapy workshops organized by the Japanese Academy of Sensory Integration (JASI) and has been used to assess children in clinical settings. In this study, the SCSIT was used to determine the presence or absence of sensory integration disorders and to understand the characteristics of the participants' sensory integration functioning. The SCSIT was scored by certified instructors of the JASI or therapists who passed the training course examination.

Developmental Coordination Disorder Questionnaire-Japanese Version (DCDQ-J)

The Developmental Coordination Disorder Questionnaire (DCDQ) is a parent questionnaire that is designed to screen children’s motor control, movement coordination, and motor skills within the context of everyday life. It has a total of 15 items and is divided into three subscales: "control during movement” (six items), “fine motor skills” (four items), and "general coordination” (five items). Higher scores indicate better coordination. We used the DCDQ-J [42] in this study. The correlation coefficient between the subscales ranged from 0.59 to 0.73, and total scores (α = 0.93) [42]. Parents scored the DCDQ-J before and after the intervention.

Canadian Occupational Performance Measure (COPM)

The COPM [43] was used to determine the children's needs for daily living, as assessed by the parents. In this study, parents were asked what they would like their children to be able to do, to clarify what was most important to the children in their daily lives. On the COPM, parents score the child’s level of performance as well as parent satisfaction with the child’s performance in daily occupations, for each item. Each item is rated on a scale of 1 to 10. In this study, the levels of performance and satisfaction were evaluated using the COPM before and after the intervention, with a mean difference of 2 or more per domain considered a clinically meaningful change.

Intervention

Ayres Sensory Integration® (ASI) intervention

The intervention group received one-to-one (therapist-child) 60-minute ASI therapy sessions twice a week for 10 weeks for a total of 20 intervention sessions. The intervention frequency was set at twice a week, considering the frequency used in previous studies and current individual intervention frequencies that are customary in Japan [27,28]. During the intervention period, participants were encouraged to continue receiving social services and to avoid receiving any new individual treatment, additional medication, or changes in their living situation.

The interventions were carried out by the first, fourth, and fifth authors who were certified instructors of the JASI or had completed all the training sessions of the certification course prior to the initiation of the study. All interveners were occupational therapists with experience working with children who had developmental disabilities in hospital-based or community-based occupational therapy clinics. For the intervention group, the characteristics of each child’s sensory integration functioning, including strengths and weaknesses (e.g., below -1 SD score items of SCSIT and informal information such as confused expression), were discussed among all the interventionists prior to the initiation of the intervention. All interventionists based their assessment findings for each child on the child’s performance on the SCSIT, the SSP, and the MABC-2. The interveners created the treatment plan and intervention strategies for each child.

Furthermore, during the intervention period, the research team had regular discussions on the intervention process to ensure the quality of the therapy. Depending on the improvement of children’s function and interests, the sort of play or toys, for example, were adjusted according to the intervention plan. Therapy sessions were conducted in a room with enough space to accommodate various therapy equipment, such as a trampoline, slide, scooter board, safety mat placed under swings, and dimmable lighting. During the intervention, the therapists spontaneously adjusted the environmental space and equipment according to the child's physical and psychological responses, expressions, interests, and reactions to specific sensory experiences, while also ensuring safety and promoting the child's achievements and competence. To record the intervention process, occupational therapists and undergraduate students were in the same therapy room, with observers maintaining an appropriate distance to avoid distracting the child or interfering with the therapy process.

Intervention Fidelity

The Ayres Sensory Integration Fidelity Measure (ASIFM) was developed to determine whether the sensory integration treatment being provided in research is in accordance with the ASI intervention, as developed and advocated by Ayres [17,44]. The ASIFM was used to confirm the fidelity and quality of the therapy that was provided to participants in this study. The ASIFM has an overall inter-rater reliability of 0.99, with inter-rater reliability ranging from 0.94 to 0.99 for each item [44]. It consists of two sections: (1) structural elements and (2) process elements. Structural elements address the therapy environment as well as the therapist's past training and qualifications to provide ASI assessment and intervention. Process elements of the ASIFM are scored by observation and rating of 10 items that address the main principles of ASI provision. This section focuses on what the therapist should be doing during the therapy session, i.e., the process of providing therapy. Items address the therapist’s attentiveness and relationship with the child, as well as the tailoring of activities to the child’s needs, and the grading of activities so that they are not too easy or too difficult for the child to perform (the “just-right challenge”). Scoring guidelines indicate that ASI fidelity for structural elements is achieved by meeting at least 85 out of 110 points. For process elements, which address the therapists’ actions during an ASI session, 80 out of 100 points are required in order for a therapy session to be confirmed as appropriately provided [18,44,45].

Data analysis

For comparison of outcome measures between the two groups, we analyzed differences in mean scores of each group using an analysis of split-plot factorial design. Furthermore, multiple comparisons using the Bonferroni method were conducted to ascertain the simple main effects between pre-post intervention in two groups, namely, the intervention and control groups. Moreover, differences between groups in the amount of change in the pre-intervention and post-intervention were assessed using the unpaired t-test and Mann-Whitney U test for outcome measure. The effect size partial η^2^ in interaction (time × group) values was interpreted as follows: small = 0.01, medium = 0.06, and large = 0.14. The effect size d in multiple comparisons was d = 0.20, indicating a small effect; d = 0.50, implying a medium effect; and d = 0.80, suggesting a large effect [46]. The Shapiro-Wilk test was used for the normal distribution with all assessment items. The t-test or Mann-Whitney U test was used to compare averages of continuous variables (such as age), and chi-square (Χ^2^) tests were used to compare the proportions of categorical variables (such as gender) between the groups. Statistical analyses were performed using SPSS software version 26 (IBM Corp., Armonk, NY) with a significance level set at 0.05.

## Results

Trial flow

**Figure 1:**
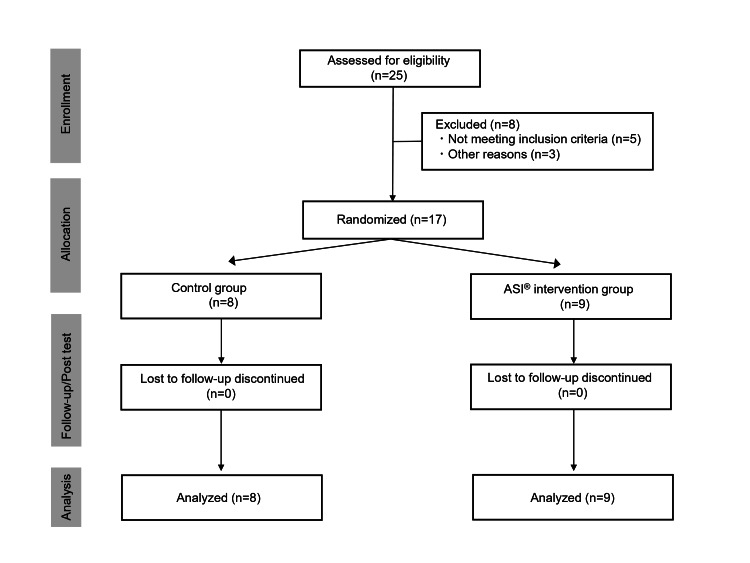
CONSORT (Consolidated Standards of Reporting Trials) diagram of participant flow throughout the study. ASI^®^: Ayres Sensory Integration.

Figure 1 depicts the research flow based on the CONSORT (Consolidated Standards of Reporting Trials) guidelines. A total of 25 participants were registered for this study. Three children did not complete the direct assessment, and five children were excluded following the exclusion criteria. Consent to participate in the study was obtained from 17 (68%) individuals who met the eligibility criteria and were randomly assigned to the ASI intervention group or control group. All participants underwent pre- and post-intervention assessments, and no subject dropped out of the study during the intervention period. In the final analysis, 17 subjects were included.

Participant Characteristics

Table 1 presents the descriptive statistics for participant characteristics and all outcome variables at baseline. Participant characteristics and pre-assessment data indicated no significant difference between the intervention group and the control group. Some participants had diagnoses in addition to DCD, e.g., ASD, ADHD, and specific learning disorder (SLD). The Mann-Whitney U test was used for the age and KSPD, t-test for SRS-2, and ADHD-RS and SSP to compare the intervention group and control group in the baseline. There were no significant differences in the number of participants, gender, age, KSPD DQ score, SRS-2, ADHD-RS, and SSP across study groups.

**Table 1:** Participant characteristics. Diagnosis shows number; KSPD shows the average of group DQ score ± SD; SRS-2, ADHD-RS, and SSP show the average score of group ± SD. DCD: developmental coordination disorder; ASD: autism spectrum disorder; ADHD: attention-deficit-hyperactivity disorder; SLD: specific learning disorder; SD: standard deviation; KSPD: Kyoto Scale of Psychological Development; DQ: developmental quotients; WISC-IV: Wechsler Intelligence Scale for Children-Fourth Edition; IQ: intelligence quotient; SRS-2: Social Responsiveness Scale-Second Edition; ADHD-RS: ADHD-Rating Scale-IV; SSP: Short Sensory Profile.

	Intervention group (n = 9)	Control group (n = 8)	p	Effect size	
				r	d
Age (month ± SD)	68.30 ± 16.07	69.99 ± 7.74	0.370	-0.23	
Range	51.42 - 106.49	55.48 - 78.33			
Sex (men: women)	8:1	6:2	0.453		
Diagnosis					
DCD	2	4			
DCD + ASD	2	3			
DCD + ADHD	2	1			
DCD + ASD + ADHD	2	-			
DCD + SLD	1	-			
KSPD: DQ (n = 16)	93.75 ± 12.48	89.0 ± 11.34	0.645	0.13	
WISC-IV Full-scale IQ (n = 1)	91	-	-		
SRS-2	51.56 ± 12.37	60.38 ± 17.736	0.248		0.58
ADHD-RS	14.22 ± 10.02	20 ± 13.34	0.325		0.49
SSP	62.25 ± 16.58	66.25 ± 25.15	0.699		0.192

Change in the primary outcome

MABC-2

**Figure 2:**
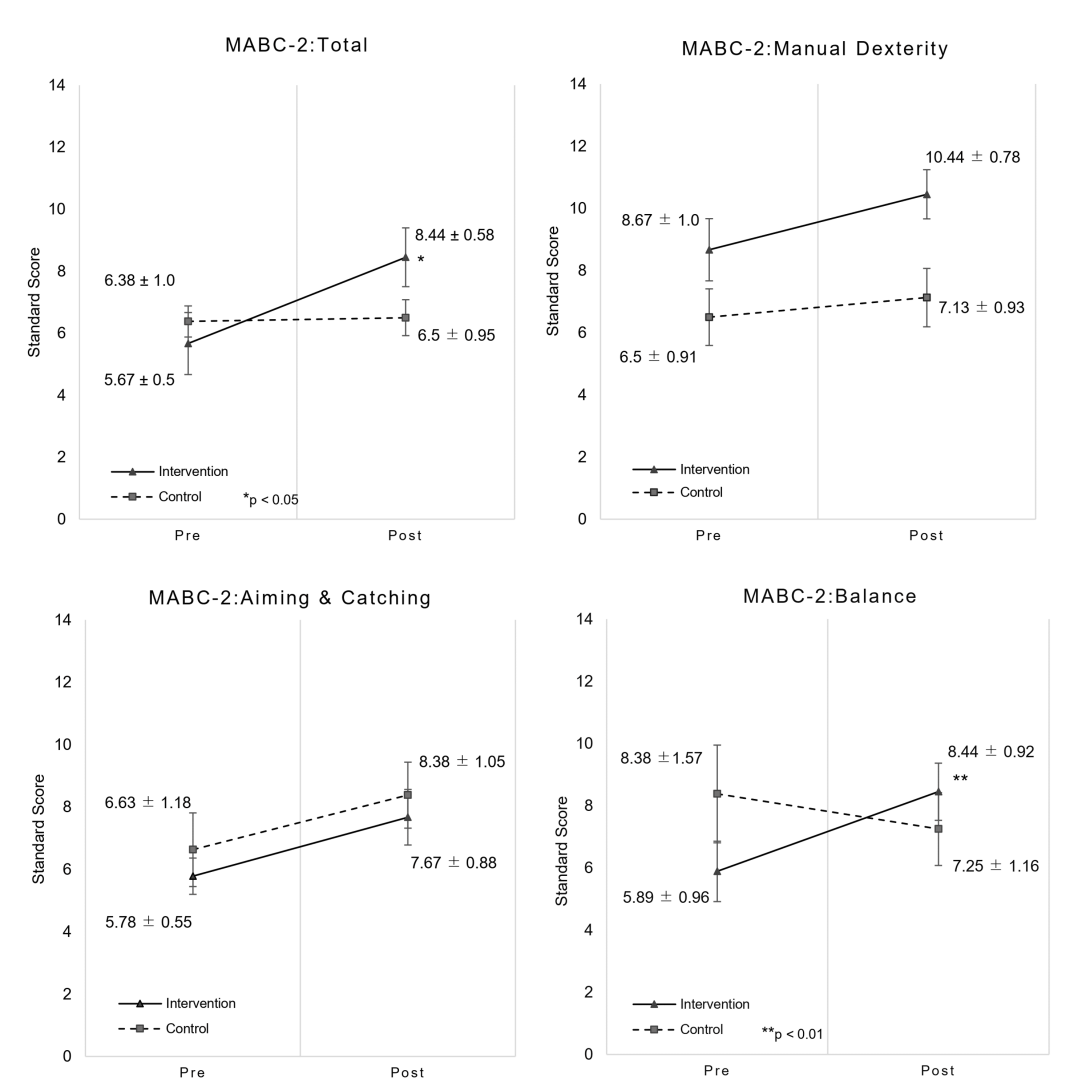
Result of time × group interaction of MABC-2. There were significant time × group interactions in the total score (p = 0.014) and the balance score (p = 0.004). The error bar represents the standard error. MABC-2 standard scores represent means and standard errors per group. MABC-2: Movement Assessment Battery for Children-Second Edition.

As shown in Figure 2, significant time × group interactions in the total scores (F (1, 15) = 7.651, p = 0.014, partial η^2^ = 0.338) and balance scores (F (1, 15) = 11.163, p = 0.004, partial η^2^ = 0.427) were observed, and a simple main effect was observed in the intervention group (total: p < 0.001, 95% CI (-3.702, -1.854), d = 1.71; balance: p = 0.002, 95% CI (-3.837, -1.274), d = 0.91) (Table 2). As for the manual dexterity score, the results of multiple comparison tests before and after the intervention showed that the simple main effect was significantly different in the intervention group (p = 0.001, 95% CI (-2.618, -0.938), d = 0.66), but the time × group interaction was not significantly different (F (1, 15) = 1.58, p = 0.228, partial η^2^ = 0.095). Similarly, significant differences were observed in the simple main effects for the aiming and catching score in the intervention group (p = 0.047, 95% CI (-3.75, -0.028), d = 0.86), while no significant differences were observed in the time × group interaction (F (1, 15) = 0.008, p = 0.932, partial η^2^ = 0.001). The amount of change of the total score in pre-and post-intervention was significantly larger in the intervention group (mean ± SD: -2.78 ± 1.20) than in the control group (mean ± SD: -0.12 ± 2.59) (p = 0.025, 95% CI (-4.89, -.416), d = -1.344). Similarly, the amount of change in the balance score in pre- and post-intervention was significantly larger in the intervention group than in the control group (p = 0.004, r = 0.69). The amount of change of the manual dexterity score in pre- and post-intervention showed no significant differences in the intervention group (mean ± SD: -1.78 ± 1.09) and the control group (mean ± SD: -0.62 ± 2.50) (p = 0.258, 95% CI (-3.306, 1.0), d = -0.611). The amount of change in the aiming and catching score in pre- and post-intervention showed no significant differences in the intervention group and the control group (p = 0.370, r = 0.24).

**Table 2:** Group comparison by primary outcome measure. Scores shown as means are GAS, group mean of T scores, and standard score for each domain of MABC-2. Outcome measure scores represent means and standard errors per group. GAS: Goal Attainment Scale; MABC-2: MABC-2: Movement Assessment Battery for Children-Second Edition; MD: manual dexterity; A&C: aiming & catching; CI: confidence interval.

Outcome variable	Group	Time period		Simple main effect (Bonferroni)			
		Pre-intervention	Post-intervention	p	95% CI		Effect size
		Mean ± SE	Mean ± SE		Lower	Upper	d
MABC-2: Total	Intervention	5.67 ± 0.5	8.44 ± 0.58	<0.001	-3.702	-1.854	1.71
	Control	6.38 ± 1.0	6.5 ± 0.95	0.895	-2.288	2.038	0.05
MABC-2: MD	Intervention	8.67 ± 1.0	10.44 ± 0.78	0.001	-2.618	-0.938	0.66
	Control	6.50 ± 0.91	7.13 ± 0.93	0.503	-2.718	1.468	0.24
MABC-2: A&C	Intervention	5.78 ± 0.55	7.67 ± 0.88	0.047	-3.75	-0.028	0.86
	Control	6.63 ± 1.18	8.38 ± 1.05	0.259	-5.116	1.616	0.55
MABC-2: Balance	Intervention	5.89 ± 0.96	8.44 ± 0.92	0.002	-3.837	-1.274	0.91
	Control	8.38 ± 1.57	7.25 ± 1.16	0.293	-1.216	3.466	0.29
GAS	Intervention	37.0 ± 0.12	63.74 ± 3.06	<0.001	-33.669	-19.82	4.12
	Control	37.73 ± 0.4	47.30 ± 3.46	0.018	-16.962	-2.188	1.38

GAS

**Figure 3:**
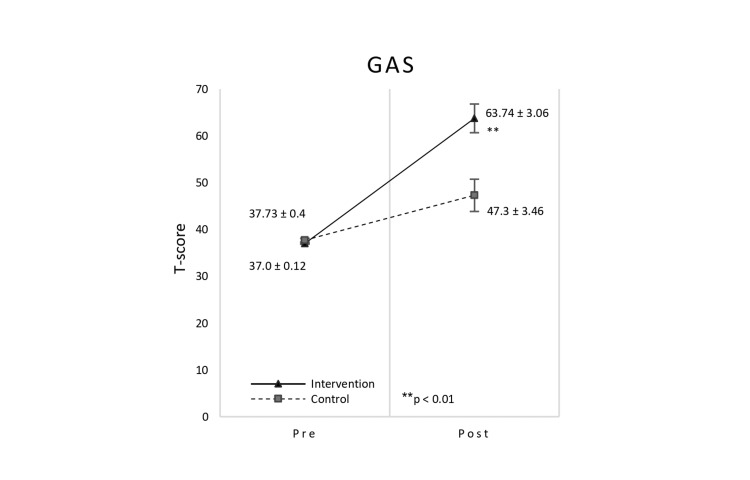
Result of time × group interaction of GAS. There was a significant time × group interaction in GAS T-score (p = 0.001). The error bar represents the standard error. Scores shown as means are GAS, group mean of T-scores. GAS: Goal Attainment Scale.

The T-score of the GAS showed a time × group interaction effect, with a significant difference in the main outcome for both the intervention and control groups (Figure 3). In the intervention group, the GAS T-score after the intervention was 63.74, whereas the control group’s GAS T-score was 47.30. In the intervention group, accordingly, the T-score was higher than 50 points, indicating a better-than-predicted achievement. The GAS had a significant time × group interaction (F (1, 15) = 15.662, p = 0.001, partial η^2^ = 0.511). The results of multiple comparison tests before and after the intervention are shown in Table 2. Both the intervention group and the control group showed significant differences in pre- and post-intervention. In addition, the amount of change in the pre- and post-intervention was significantly larger in the intervention group (mean ± SD: -26.74 ± 9.0) than in the control group (mean ± SD: -9.57 ± 8.84) (p = 0.001, 95% CI (-26.41, -7.92), d = -1.923). Table 3 depicts the breakdown of the GAS goals set with the parents of participants. Goals were classified based on the International Classification of Functioning, Disability, and Health (ICF), following previous study analysis [19]. The goals listed in GAS were classified into ICF function level and activities level. The chi-square test was conducted between the two groups. There was no significant difference between the two groups in the number of categories (p = 0.606).

**Table 3:** Goal set out of GAS. The numbers refer to the number of children. GAS: Goal Attainment Scale.

Type of goal	Intervention group (n = 9)	Control group (n = 8)
Challenge/behavior modification	6	7
Posture control	4	3
Fine motor (chopsticks, spoon, scissors, pinch)	4	3
Play (rope jumping, origami, bicycle, pole climbing, etc.)	1	5
Sport (dancing, catching ball, throwing ball)	4	0
Gross motor (jumping, hopping)	2	2
Writing letter	1	1
Communication	1	0
Sleep	1	0
Emotional regulation	1	0
Others	1	1

Change in the secondary outcome

**Figure 4:**
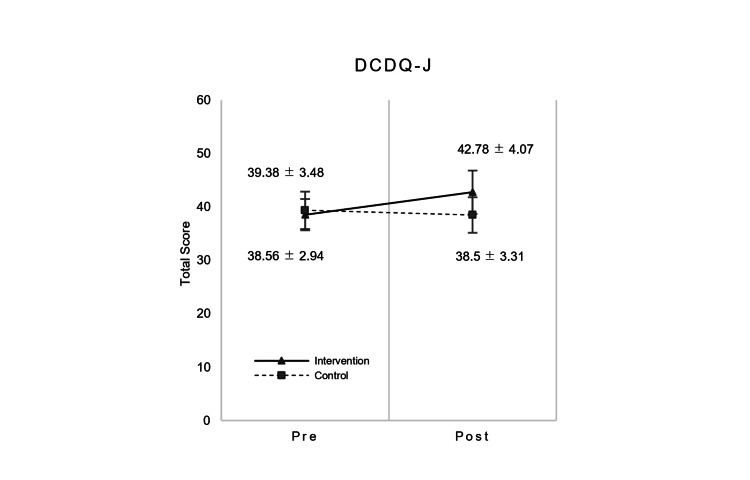
Result of time × group interaction of DCDQ-J. DCDQ-J total scores represent means and standard errors per group. DCDQ-J: The Japanese version of the Developmental Coordination Disorder Questionnaire.

The performance and satisfaction scores of COPM showed significant differences before and after the intervention period for the main effect, but no time × group interaction effects were found (Table 4). No significant differences between pre- and post-intervention interactions and main effects were found for the DCDQ-J (F (1, 15) = 1.148, p = 0.301, partial η^2^ = 0.071) (Table 4 and Figure 4). The amount of change in the DCDQ-J score in pre- and post-intervention had no significant difference in the intervention group (mean ± SD: -4.22 ± 5.65) and in the control group (mean ± SD: 0.88 ± 13) (p = 0.331, 95% CI (-16.275, 6.081), d = -0.521). Furthermore, three of the SCSIT items demonstrated an interaction: finger identification (p = 0.046), standing balance with eyes open (p = 0.016), and motor accuracy-revised - right (p = 0.038). One item showed a simple main effect in the intervention group (motor accuracy-revised - right (p = 0.044)), and 13 items showed no significant differences in both time × group interaction and simple main effects.

**Table 4:** Result of COPM and DCDQ-J. Outcome measure scores represent means and standard errors per group. Scores shown as means are COPM, the mean of each domain; and DCDQ-J, the mean of the overall score. COPM: Canadian Occupational Performance Measure; DCDQ-J: The Japanese version of the Developmental Coordination Disorder Questionnaire; df: degree of freedom; CI: confidence interval.

Outcome variable	Group	Time period		Interaction (time × group)				Simple main effect (Bonferroni)			
												Pre-intervention	Post-intervention	F	p	df	Effect size	p	95% CI		Effect size
		Mean ± SE	Mean ± SE	Partial η^2^	Lower	Upper	d											
		COPM: Performance	Intervention	4.36 ± 0.31	5.91 ± 0.35	1.453	0.247	(1, 15)	0.088	0.015	-2.721	-0.39	1.58
Control	3.19 ± 0.40		5.66 ± 0.46	0.003	-3.813					-1.125	2.01
COPM: Satisfaction	Intervention	4.07 ± 0.39	5.89 ± 0.44	1.749	0.206	(1, 15)	0.104	0.002	-2.733	-0.923	1.46
	Control	3.36 ± 0.46	6.19 ± 0.65					0.004	-4.433	-1.234	1.78
DCDQ-J	Intervention	38.56 ± 2.94	42.78 ± 4.07	1.148	0.301	(1, 15)	0.071	0.055	-8.567	0.122	0.4
	Control	39.38 ± 3.48	38.5 ± 3.31					0.854	-9.993	11.743	0.09

Fidelity to Intervention

The 10th author who participated in the development of ASIFM assessed the treatment session video data (initial sessions and 19th or 20th sessions for each participating child) using the ASIFM. The ASIFM average score was 96.49 (range = 92.7-99.3). Thus, all treatment sessions met the criteria for an ASI intervention.

Harms

There were no adverse events at any point in this study.

## Discussion

This study presents an RCT of ASI intervention for children with DCD to examine whether ASI is likely to promote improvements in these children’s motor coordination. In Japan, occupational therapy interventions have focused on coordination tasks, but their effects have not been reported. This is the first report of the RCT on ASI for children with DCD in Japan, and perhaps internationally. The results of this study suggest that ASI intervention for children with DCD improves motor coordination and helps them achieve their daily activity goals.

In this study, it is demonstrated that ASI intervention has shown a significant effect on motor coordination function. The total score and balance score of MABC-2 and GAS showed dramatic improvement in interaction and simple main effect with the intervention group. Before intervention in this study, all assessment data and information from parents about their children's daily lives were collated and interpreted to create an intervention plan that was formulated for each child. In this study, eight out of nine children had an MABC-2 balance standard score of less than 10. Also, six out of nine children were below the 16th percentile score. Furthermore, out of the nine children who were assigned to the intervention group, five children scored -1.0 SD or less (i.e., a score of -1.1 or lower) on the SCSIT standing balance in both eyes open and eyes closed test. It is indicated that approximately half of the children had great difficulty with balance functions. It has been reported that children with DCD have difficulty with postural adjustment and balance function [47,48]. It is likely that many participating children in the intervention group showed difficulty with balance abilities. As a result, during the initial period of the intervention, we focused on play that requires balancing skills, which was a challenge for most of the children. For example, children who were sensitive to changes in head position and reacted fearfully to vestibular sensations were placed in situations that required simple postural balance adjustments, such as crawling up an inclined surface or maintaining balance while seated on a suspended platform that swings back and forth or gently rotates. It has been proven that the need for postural control on unstable scaffolding influences deep touch-pressure sensations that contribute to body awareness and refinement of motor skills [49], and the opportunity of experiences with vestibular and proprioceptive sensations is important [17,50] for refinement of balance. In particular, we devised methods to immerse the child in play within the treatment setting. For instance, we created a story in which children could play as their favorite characters and challenged them to a treasure hunt. In this way, the children could actively and repeatedly be challenged by the therapy, feeling the feedback of sensory information within themselves and from the environment, so as to improve their postural control functions, which led to the improvement of the balance score and the total score of MABC-2, and the one-leg balance score of SCSIT.

Furthermore, this study showed ASI intervention improved children’s daily activities. After completion of the intervention program, the mean GAS T-score was 63.74 for the intervention group and 47.3 for the control group, indicating that the intervention improved motor skills significantly. As in previous studies on the effectiveness of ASI intervention [19], the study’s results this time also indicated significantly improved GAS scores in the intervention group. In the intervention group, the children were given an individualized program, and the therapy environment was set up according to the characteristics of the children. Goals related to physical functions, such as maintaining posture, gaining strength, throwing a ball, and catching a ball (Table 3), were set up along with the parents for the participating children. Other goals related to personal intention and motivation, such as "to try two physical games and engage in them" and "to respond to invitations" were also established. The children in the intervention group were able to perform gross motor skills and posture adjustments, such as jumping on one foot, jumping from blocks, crawling across bridges, and adjusting their posture during play and mealtimes, showing results that exceeded expectations. In the therapy settings, children were repeatedly observed trying other new tasks and playing. We believe this highly emphasized individualization, which is essential in the provision of ASI intervention, gave the children confidence and enabled them to challenge similar tasks and attempt to participate in playing and new activities in their daily lives. The therapist devises an environment and flexibly creates a fun and playful “just right challenge” setting for the child while observing the child's reactions to the situation, interests, and comments. Due to the importance of individualization in ASI, the ambiguity and reproducibility of the intervention have been questioned. In all video-recorded intervention sessions, the fidelity scores were 90 or higher, confirming that we were able to provide therapy based on ASI principles, which led to improvements in children's motor performance, increased motivation, and improved GAS scores. In future studies, it will continue to be important to use such objective criteria to clearly identify the characteristics of interventions that can be tailored to the individual needs of subjects with diverse conditions.

However, regarding aiming and catching and manual dexterity of MABC-2, no significant difference was found in the time × group interaction while the simple main effect did present improvement with the intervention group. In terms of throwing and catching skill and fine motor functions (e.g., aiming and catching and manual dexterity of MABC-2), it showcases not only the motor function of hands, but the bilateral coordination, spatial awareness, or eye-hand coordination function are also taken into account. A previous study showed that there was no significant difference in the manual dexterity score of MABC-2 in small-group intervention promoting the development of eye-hand coordination and fine finger movement during play activities. On the other hand, it was pointed out that the manual dexterity score improved significantly immediately after the intervention when compared between children at risk of motor impairment and typical development children, but the improvement did not last long [51]. Accordingly, to obtain a stronger effect, further research is needed to consider posture control function, intervention period, and intervention methods. However, the GAS T-score obviously improved after intervention in the intervention group. As GAS is more individual-oriented, the actual and minimal improvements are more easily reflected than standardized assessment. In this study, "challenge/behavior modification" (Table 3) was the most frequent goal participants mentioned. The improvement of GAS results could show that children’s motivation to join challenging activities was increased in the therapy session. In addition, those changes could affect the positive attitude by joining various activities, as shown in Table 3.

While the secondary assessments were administered, the DCDQ-J and COPM scores indicated no interactions with the intervention, nor any significant changes from the initial intervention session to the final session. The needs identified by the COPM ranged from simple motor skills, such as being able to keep good posture while seated, to more complex actions such as playing "jumping rope," to emotional and communication abilities, such as "smooth communication with others” and "able to control feelings." Improvements in motor performance and movement-related emotional reactions were easily observed by the end of data collection. However, other important functions, such as higher-order self-regulation and communication skills, were not measured in this study, and may not have been affected by the intervention. In addition, GAS has clear criteria for setting and scoring specific, observable goals, whereas the COPM is entirely dependent upon parents’ subjective judgments for scoring. Consequently, it may be that the subjectivity of the COPM scoring resulted in biased findings. Similarly, the DCDQ-J score improved in the intervention group although the result also did not show a statistically significant interaction between the two groups. A previous study [52] bears the same result. DCDQ-J is a questionnaire about comprehensive coordination in activities of daily living other than specific individual motor activities when compared with MABC-2, and it appears the score is subjective to the respondent, i.e., parents’ judgment. Therefore, no significant difference in score between the two groups is shown in this case.

From the result, ASI intervention could show an effective approach to deficits in the internal model. The children improved the speed of their posture control and the variety of movements to adjust to the surrounding environment during the therapy sessions. Children with DCD have sensory processing impairments, which are associated with poor motor coordination [53]. If the body schema remains ambiguous, movement cannot be executed accurately [9,10]. In case sensory information from the results of that movement is not properly feed-backed, errors may occur, and movement is less likely to be modified. Since the participants in this study also had low somatosensory scores on the SCSIT, it is believed that they were unable to receive and properly process sensory information to create a body schema and body image, leading to difficulties in motor coordination. Reduced postural balance, difficulty maintaining posture, and immaturity of postural reflexes were also observed. (e.g., children have difficulty maintaining the sitting posture). For this reason, the therapy focused on providing proprioceptive, tactile, and vestibular sensory information, which are important for posture control and visuomotor abilities, praxis, and motor planning [17]. We then gradually increased the level of difficulty for each child, setting up play situations in which the child had to perform actions such as jumping at the right moment to avoid the rolling poll on the trampoline, instantly matching the distance in space with his or her own physical information. This approach allowed the children to update their body schema, which may have led to changes in their performance. However, since we did not conduct an evaluation that truly reflected the updating of the body schema, we believe it will be necessary to combine such experiments in the future.

Future research should examine the effects of frequency of intervention, the degree of maintenance of effects after cessation of the intervention, and the effects on daily life after receiving ASI intervention, to provide guidelines for optimal session times and duration of this intervention. We gathered results that indicated improvements in the coordinated movement abilities of children with DCD after 20 ASI intervention sessions that were provided twice a week, over a 10-week period. The intervention frequency in a previously published RCT study for children with autism was three times per week [19]. Furthermore, we anticipate to expand our understanding of DCD and ultimately to support the development of motor skills for these children. Additional research goals to pursue may include clarifying the presence or absence of changes in specific movement skills over time, and determining what the predisposing factors of movement difficulties might be. These deeper understandings of the nature of DCD will help professionals to provide rehabilitation services precisely tailored to meet the specific needs of each child with DCD, within the contexts of the child’s interests, the child’s family, and the child’s community.

Limitations

Blinding of the parents was not possible, although blinding of the assessors was done in practice. In this study, we requested parents to keep their usual daily life regardless of the intervention. However, there is a possibility of bias in parents in the intervention group, since the parents themselves knew whether or not their child had received the intervention. The outcome measure parents responded to may be influenced. In addition, SCSIT developed by A. Jean Ayres in the 1960s [26] was from data recruited in the United States, and the standardized data with Japanese participants had not been examined. That condition may affect the interpretation of the result. Furthermore, the sample size in this study was small with each group. Small samples may not adequately capture the variability within a population, leading to potential biases or limited statistical power. Although there are limitations in recruiting suitable participants in practical clinical conditions, future studies need to consider larger samples.

In the future, it very likely will continue to be necessary for occupational and physical therapists to work toward clarifying the impacts of sensory integration, cognition, and emotion regulation on several aspects of movement. For example, our profession needs to pursue a deeper understanding of ideation (the cognitive process of generating a movement goal), motor planning (the cognitive ability to plan the sequence of actions needed to attain the movement goal), and performance (the actual performance of movements to attain the goal). Ultimately, it is hoped that a deeper understanding of the causes of DCD will lead to highly efficacious interventions that are individually tailored for children with various types of DCD.

## Conclusions

This is the first RCT to demonstrate that ASI® intervention can improve motor coordination difficulties in children with DCD. In addition, the findings may enhance daily activities and motivation. This finding provides for evidence-based practice for children with DCD. On the other hand, as self-regulation and communication skills are more complicated in daily life, this study has shown limited results. Further research has to be focused on investigating intervention frequency, long-term effectiveness, and influence of parents, which will lead to more effective interventions for children with developmental disorders.

## Appendices

**Table 5:** Goal Attainment Scaling form. Attainment level: -2: much less than expected; -1: somewhat less than expected; 0: expected level of attainment; +1: somewhat more than expected; +2: much more than expected.

	Goal 1	Goal 2	Goal 3
Importance			
Weight			
+ 2			
				+ 1			
0							
				- 1			
- 2							
